# Diagnostic accuracy of the Geneva clinical scale for diagnostic prediction of pulmonary embolism in adults aged 18 and older admitted between 2009 and 2020 with suspected pulmonary embolism at a Third-Level Institution in Colombia: A retrospective cohort study

**DOI:** 10.1097/MD.0000000000041603

**Published:** 2025-02-28

**Authors:** Cristian López-Vega, Michel Pérez-Garzón, Leonora Ortiz-García-Herreros, Alirio Bastidas-Goyes, Manuel Aramendiz-Narvaez, Estefan Ramos-Isaza, Henry Robayo-Amortegui

**Affiliations:** a Intensive Care, Clínica Universidad de La Sabana, Chía, Colombia; b Department of Critical Care Medicine, Extracorporeal Life Support Unit (USVEC), Fundación Clínica Shaio, Bogotá D.C., Colombia; c Department of Critical Medicine and Intensive Care, Fundación Clínica Shaio, Bogotá, Colombia; d School of Medicine, Universidad de La Sabana, Chía, Cundinamarca, Colombia; e Radiology Resident, Universidad de La Sabana, Chía, Cundinamarca, Colombia.

**Keywords:** computed tomography pulmonay angiographic, diagnosis, Geneva original score, pulmonary embolism, standardized clinical decision

## Abstract

To assess the overall applicability of the Geneva scale for diagnosing pulmonary embolism in adults aged 18 and older. A retrospective cohort study with diagnostic test analysis was conducted on patients in the emergency department or hospitalized between 2009 and 2020 with suspected pulmonary embolism at a Third-Level Institution in Colombia. Local study. The original and simplified Geneva scores were applied to 1237 subjects aged 18 and older with suspected pulmonary embolism and compared with confirmatory results from pulmonary angiography. All necessary variables for constructing the original and simplified Geneva rules were recorded, and calculations for sensitivity (S), specificity (E), likelihood ratios, and receiver operating characteristic curves were performed. The Geneva original score exhibited an S, E, positive likelihood ratio, negative likelihood ratio, and area under the curve of 60%, 54%, 1.3, 0.728, and 0.506, respectively. The simplified Geneva score showed 59%, 57%, 1.4, 0.7, and 0.546 for S, E, positive likelihood ratio, negative likelihood ratio, and area under the curve, respectively. The use of the original or simplified Geneva score in our population may not be useful for a diagnostic approach to pulmonary embolism. Both scales demonstrate almost negligible discriminatory capacity, necessitating the evaluation of other standardized clinical decision rules to assess the diagnosis and pretest probability of pulmonary thromboembolism.

## 1. Introduction

Venous embolism stands as the third most frequent cause of cardiovascular-related deaths, a phenomenon gaining increasing significance in aging populations.^[1]^ Specifically, pulmonary embolism (PE) involves the lodging of a thrombus in one of the pulmonary arteries. While this thrombus can originate from various venous pathways, it most commonly arises from the deep venous system of the lower limbs,^[2]^ linking to higher mortality due to adaptive phenomena like pulmonary hypertension and right heart failure.^[3]^

Mortality rates have been extensively documented in high-income countries, with limited studies in the Latin American region. In Colombia, a 2008 study by Dennis et al reported a hospital mortality rate of 14.8%, associated with hip fracture and hypotension during admission.^[4]^

Clinical presentation and symptoms are nonspecific; however, a combination of symptom awareness and predisposing factors determines the likelihood of encountering this condition.^[5]^ Probability is established once diagnostic imaging, confirming PE through pulmonary angiography (CPTA), is considered. It is crucial to note that no isolated test provides sufficient diagnostic performance to conclusively rule in or rule out the diagnosis. Instead, a diagnosis is reached by amalgamating clinical findings with paraclinical and imaging results.^[2]^

Standardized clinical decision rules (CDRs) have been formulated to establish this pretest probability. These instruments incorporate variables extracted from medical history, physical examinations, and diagnostic tests to quantify the probability of a clinical diagnosis, prognosis, or response to treatment in each individual patient.^[6]^ For diagnosing pulmonary embolism, noteworthy CDRs include the Wells and Geneva scales, both of which have been simplified from their original versions and validated in diverse populations.^[5,7,8]^

The original Geneva scale, developed in 2006 through logistic regression analysis,^[6]^ is a CDR based on objective clinical elements, encompassing 8 independent variables with different weights contributing to the final result. To address potential calculation errors, a simplified version was derived, assigning equal weight to different variables.^[7,9]^ The exception is a double weighting for a heart rate exceeding 95 beats per minute (BPM). This simplified scale has been validated, demonstrating the possibility of simplifying the original scale without compromising diagnostic acuity.^[6,7]^ Both scales classify clinical probability based on the score into mild, moderate, and severe categories, or probable and improbable cases of thromboembolism.

The primary objective of this study was to evaluate the general applicability of the Geneva scale for diagnosing PE in adults aged 18 and older admitted with suspected PE to a Third-Level Institution in Colombia. Secondary objectives included characterizing the population with suspected PE based on clinical probability, risk factors, and established diagnostic criteria, as well as determining diagnostic performance applied to a Third-Level Institution.

## 2. Methods

**Figure 1.**
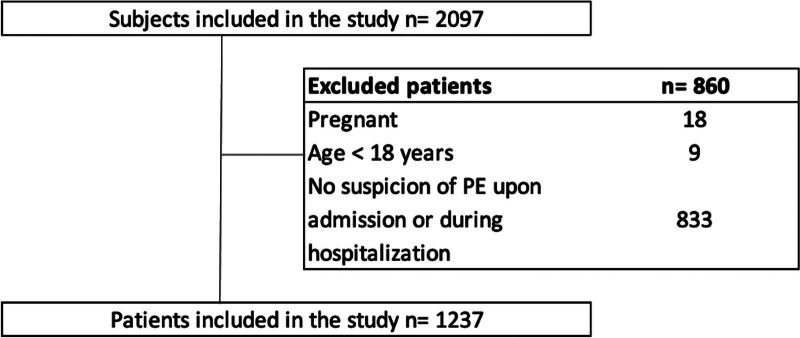
Flowchart of the admission of subjects to the study.

A retrospective cohort study with diagnostic test analysis was conducted on subjects aged 18 and older between 2009 and 2020 at a Third-Level University Clinic. The study was approved by the Ethics Committee of Clínica Universidad de La Sabana (Code #20220501) carried out in accordance with the modified Declaration of Helsinki, and informed consent was waived according to its retrospective nature and local regulations. Participants were those presenting with suspected PE and subsequently undergoing computed tomography pulmonary angiographic (CTPA) in the emergency and hospitalization services at Clínica Universidad de La Sabana, a third-level care center. All subjects aged 18 and older with suspected PE, a documented CT angiography report, and complete information for calculating both the original and simplified Geneva scales (as illustrated in Fig. 1) were included. Exclusion criteria encompassed individuals under 18 years old, pregnant women, patients without suspected PE upon admission to the emergency or hospitalization, and subjects with incomplete medical histories.

Variables such as age and gender, location of disease suspicion (emergency or hospitalization), history of surgery in the last 4 weeks, use of general anesthesia, lower limb fracture, malignancy, active cancer within the last year, clinical findings of immobilization exceeding 3 days, dyspnea, duration of dyspnea, chest pain, hemoptysis, signs and symptoms of deep vein thrombosis, the most probable PE diagnosis, heart rate, whether a CTPA was performed, and CTPA result for PE by the radiology department were independently included.

Each score was calculated numerically and subsequently categorized based on probability. The revised Geneva score was classified into 3 levels (low: 0–3, moderate: 4–10, and high: 11–22).^[6]^ The diagnosis of PE was established using the CTPA result interpreted by the institutional radiology service as a positive study for PE.

Clinical data extracted from medical records were collected using the RedCap electronic data capture software. Subsequently, the data were imported into an Excel spreadsheet and analyzed using the R-Studio statistical program. An initial review of the data for each variable was conducted to assess the percentage of missing values, which should not exceed 20%. Qualitative variables were summarized in terms of frequencies and percentages, while quantitative variables were presented based on their distribution—normality using mean and standard deviation, and non-normality using median and interquartile range. Bivariate analysis was performed with each studied variable, employing the Chi-square test as a parametric test for hypothesis testing.

Following the construction of scores, a receiver operating characteristic curve (ROC) analysis was conducted for each scale, calculating the AUC with a 95% of confidence interval. A statistically significant *P*-value was set at <.05. The diagnostic performance of the scales was compared by assessing the AUC. The interpretation of AUC values adhered to the following criteria: <0.5: no predictive capacity, 0.6 to 0.69: weak predictive capacity, 0.7 to 0.79: acceptable predictive capacity, 0.8 to 0.89: excellent predictive capacity, 0.9: outstanding predictive capacity.

The study followed the ethical recommendations of the Helsinki Declaration and Resolution 8430 of 1993 for human research, ensuring data confidentiality and protection. The research protocol received approval from the ethics committee of Clínica Universidad de La Sabana, with approval number 20220501.

Sample size calculation aligned with validation studies for each scale,^[6,7]^ aiming for a power of 0.90 and a significance level of 0.05, Yates-corrected. The total sample size was set at 1284 patients, considering a sensitivity (S) of 90% to 99%, specificity (E) of 27% to 34%, and a prevalence of 27% for the original Geneva scale with an AUC of 0.73. For the simplified Geneva scale, an AUC of 0.74 was anticipated.^[10]^ Finally, Microsoft Excel 2016 (Microsoft Corporation, Redmond, WA), RedCAP (Vanderbilt University, Nashville, TN), and R-Studio were utilized for statistical analysis.

## 3. Results

From the sample collected between 2009 and 2020, information from 2098 patients was gathered. Among these, 1237 met the inclusion criteria, constituting 58% of the collected sample (Fig. 1). The mean age was 60.49 with a standard deviation of 19.38, and a majority of patients were female (52.8%). 860 patients were excluded, representing 42% of the initial sample. Among them, 18 were pregnant, 9 were under 18 years old, and 830 lacked suspicion or clinical signs of PE upon admission or during hospitalization. Out of the excluded, 488 patients (39%) were confirmed with a PE diagnosis by CTPA. The description of the study population, including age and gender independently of variables in both the original and simplified Geneva scores, is presented in Table 1, differentiating findings for the general sample and confirmation or rejection of PE diagnosis by CTPA.

**Table 1:** Clinical and demographic characteristics of 1237 patients included.

Variable	All patients (n) 1237	Percentage (%)	Diagnosis of PE by CTPA (n) 488	Percentage (%)	PE excluded by CTPA (n) 749	Percentage (%)
Age (SD)	60.49	19.38%	61.48	19.54%	59.84	19.26%
Female, n (%)	653	52.8%	253	51.8%	400	53.4%
Age >65 yr, n (%)	614	49.6%	263	53.9%	351	46.9%
History of PE/DVT, n (%)	189	15.3%	79	16.2%	110	14.7%
Surgery or fracture in the last month	275	22.2%	113	23.2%	162	21.6%
Malignancy, n (%)	123	9.9%	54	11.1%	69	9.2%
Unilateral leg pain, n (%)	182	14.7%	80	16.4%	102	13.6%
Hemoptysis, n (%)	71	5.7%	37	7.6%	34	4.5%
Heart rate 75–94 bpm, n (%)	480	38.8%	195	40.0%	285	38.1%
Heart rate ≥95 bpm, n (%)	527	42.6%	230	47.1%	297	39.7%
Edema or tenderness, n (%)	186	15.0%	81	16.6%	105	14.0%

BPM = beats per minute, CTPA = computed tomography pulmonay angiographic, DVT = deep vein thrombosis, EP = pulmonary embolism, SD = standard deviation.

Analyzing patient characteristics based on the CDRs, the Geneva score, reveals that the most prevalent variable in our population is age over 65 years (49.6%), followed by a heart rate exceeding 95 bpm (42.6%). Variables with lower proportions in the general sample include hemoptysis (5.7%) and malignancy (9.9%). These findings align with patients whose PE diagnosis was confirmed by CTPA, with 53.9% and 47.1% having age over 65 and a heart rate exceeding 95 bpm, respectively. Similarly, lower frequency variables in both groups are hemoptysis (7.6%) and malignancy (11.1%).

Furthermore, an evaluation of the performance of individual variables in the Geneva score (see Table 2) indicates that, in general, each variable independently has a S lower than 50%, except for age over 65 years, which exhibits a S of 54%, falling below the expected threshold for a CDR. Conversely, the highest E values are for hemoptysis (95%) and malignancy (91%), while most other variables exhibit higher E than S, except for age over 65 years, whose values are closely aligned. The positive predictive values range from 41% to 52%, similar to negative predictive values, most of which are around 61%. Overall accuracy for each variable independently is >0.5, with hemoptysis being the only 1 exceeding 0.6. Regarding likelihood ratio, the positive values range between 1 and 1.6, with hemoptysis standing out as the highest. The negative values are below 1, indicating appropriateness of the test. Independently, each variable shows a prevalence of over 40% for PE, with the highest being 52% in patients with hemoptysis. Upon evaluating different variables, the alternative hypothesis was accepted for age over 65 years, hemoptysis, and heart rate exceeding 95 bpm in patients with PE with a *P*-value < .05.

**Table 2:** Epidemiological measures of Geneva score variables.

Variable	S^1^ (%)	E^2^ (%)	RR^3^	VPP^4^ (%)	VPN^5^ (%)	LR+^6^	LR‐^7^	Prevalence	*P*
Age >65 yr	54%	53%	1.186	43%	64%	1.15	0.8677	43%	.005
History of PE/DVT	16%	85%	1.071	42%	61%	1.102	0.9824	42%	.4336
Surgery or fracture in the last month	23%	78%	1.054	41%	61%	1.071	0.9805	41%	.4732
Malignancy	11%	91%	1.127	44%	61%	1.201	0.9796	44%	.2609
Unilateral leg pain	16%	86%	1.137	44%	61%	1.204	0.9679	44%	.1433
Hemoptysis	8%	95%	1.347	52%	61%	1.67	0.9681	52%	.0201
Heart rate 75–94 bpm	40%	62%	1.05	41%	61%	1.05	0.9692	41%	.3869
Heart rate ≥95 bpm	47%	60%	1.201	44%	64%	1.189	0.8761	44%	.0005
Edema or tenderness	17%	86%	1.125	44%	61%	1.184	0.97	44%	.1769

BPM = beats per minute, DVT = deep vein thrombosis, E = specificity, LR‐ = negative likelihood ratio, LR+ = positive likelihood ratio, PE = pulmonary embolism, RR = relative risk, S = sensitivity, VPN = negative predictive value, VPP = positive predictive value.

Concerning patient classification based on both scales, patients were organized according to the obtained score in 3 or 2 levels. For the original Geneva score in 3 levels, 22.2% had a low probability, 66.1% moderate, and 11.7% high (Table 3). The proportion of patients with pulmonary embolism for each risk group was 15.6% for low, 70.7% for moderate, and 13.7% for high. When classified into 2 levels, 51.5% were categorized as probable, and 48.5% as not probable. Similarly, of the 488 patients confirmed with a diagnosis by CTPA, 60.5% had been classified as probable, and 39.5% as not probable.

**Table 3:** Description of Geneva original and simplified score calculation.

The Geneva original score										
	Low			Moderate		High			Total	
Patients, n (%)	274	22.2%		818	66.1%	145	11.7%		1237	100%
Patients with confirmed PE, n (%)	76	15.6%		345	70.7%	67	13.7%		488	
	**Probable**				**Not probable**				**Total**	
Patients, n (%)	637		51.5%		600	48.5%			1237	
Patients with confirmed PE, n (%)	295		60.5%		193	39.5%			488	

Simplified Geneva score										
	Low			Moderate		High			Total	
Patients, n (%)	244	19.7%		887	71.7%	145		11.7%	1237	100%
Patients with confirmed PE, n (%)	68	13.9%		370	75.8%	50		10.2%	488	100%
	**Probable**				**Not probable**				**Total**	
Patients, n (%)	609		49.2%		628	50.8%			1237	100%
Patients with confirmed PE, n (%)	288		59.0%		200	41.0%			1237	100%

EP = pulmonary embolism.

Furthermore, in the case of the simplified scale, when classified into 3 levels, the distribution of patients was 19.7%, 71.7%, and 11.7% for low, moderate, and high, respectively, in the total sample. Of these, 13.9% of those considered low probability had their pulmonary embolism diagnosis confirmed, 75.8% for moderate, and 10.2% for high probability. When classified into 2 levels, 49.2% were deemed probable, and 50.8% not probable. In cases where the diagnosis was confirmed, 59% were classified as probable and 41% as not probable.

Therefore, when evaluating the performance of the scales in classifying patients into 2 levels, the original scale exhibits a S of 60% and E of 54%, with a positive predictive value of 0.463 and negative predictive value of 0.678. This closely mirrors the findings in the simplified scale, where S is 59%, E is 57%, and positive and negative predictive values are 0.473 and 0.678, respectively (Table 4).

**Table 4:** Dichotomous results of Geneva original and simplified score.

Variable	S (%)	E (%)	RR	VPP	VPN	LR+	LR‐	*P*
Original	60	54	1.44	0.463	0.678	1324	0.728	<.001
Simplified	59	57	1.48	0.473	0.682	1377	0.717	<.001

E = specificity, LR‐ = negative likelihood ratio, LR+ = positive likelihood ratio, RR = relative risk, S = sensitivity, VPN = negative predictive value, VPP = positive predictive value.

**Figure 2.**
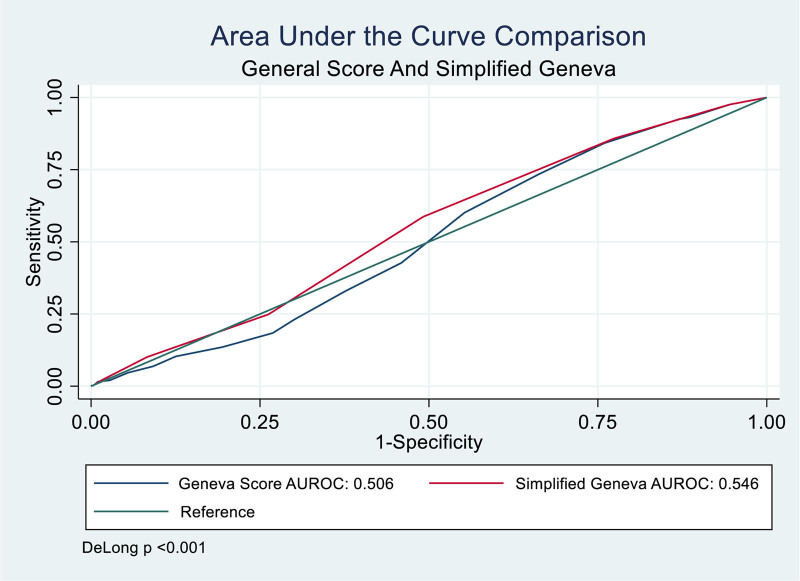
Area under the curve comparison.

Additionally, to compare the diagnostic performance of both scales, AUC were calculated (Table 5), yielding an AUC of 0.506 with a CI 95% between 0.479 and 0.533 for the original Geneva scale and 0.546 with a IC 95% between 0.519 and 0.573 for the simplified Geneva scale. Evaluating the difference between the AUCs yielded a *P*-value of .30, making it impossible to reject the null hypothesis, as depicted in Figure 2.

**Table 5:** Area under the curve comparison.

Escala	AUC	CI 95%
Geneva original	0.506	0.479–0.533
Geneva simplified	0.546	0.519–0.573

AUC = area under the curve, CI = confidence interval.

## 4. Discussion

The main objective of this study was to evaluate the diagnostic accuracy of the Geneva scale in adults aged 18 and above with suspected PE, considering that the assessed variables are easily applicable due to the objective clinical items they evaluate. This was contrasted with the CTPA report to determine the validity of the scales.

The results obtained in this study differ from previous studies, notably in terms of diagnostic accuracy values. Compared to the original study by Le Gal et al,^[6]^ which focused on an emergency department population, our study included both emergency and hospitalized patients. Additionally, the prevalence of PE was lower in the study population (23% vs 39%) in comparison to ours. The AUC for the group evaluated by Le Gal was 0.74, while in our study, it was 0.506. Another study by Klok et al^[11]^ reported a PE prevalence of 16%, with an AUC of 0.73 for the scale in that group.

In specific populations such as pregnant women, although the incidence of PE is low, its significant impact is due to the associated high mortality. Diagnosing PE in this special population is fraught with challenges due to physiological changes during pregnancy and the lack of clinically validated decision-making rules for these patients. The use of the Geneva and revised Geneva scales in pregnant women has shown low correlation between risk categories and confirmed pathology (kappa correlation index of 0.154 and a 17% prevalence in the low-risk group).^[12]^ Studies have been conducted to develop diagnostic algorithms integrating clinical decision rules, such as the modified Geneva score and the YEARS scale. The 2018 CT PE study validated an algorithm incorporating the revised Geneva scale, d-dimer, and lower limb compression ultrasonography, proving its utility in ruling out the disease and correctly classifying patients requiring treatment for pulmonary thromboembolism. Similarly, the 2019 ARTEMIS study used the YEARS algorithm (adapted for pregnancy) with d-dimer and compression ultrasonography, demonstrating good discriminatory performance in ruling out PE and reducing the need for confirmatory imaging tests.^[13,14]^ A version of the Geneva scale adapted for pregnancy has been validated, showing an improvement in the ROC curve area (0.795 for the modified scale versus 0.684 for the revised Geneva scale), emphasizing the need for its integration into a comprehensive diagnostic algorithm that includes additional tests to ensure safe decision-making.^[15]^ Finally, for the elderly population, the Wells scale performs better compared to the Geneva scale, particularly when used in conjunction with d-dimer testing to enhance diagnostic exclusion of PE.^[16]^

Bastidas et al previously conducted a validation of 3 CDRs for our population, documenting an AUC for the original Geneva score of 0.6, slightly better than our study.^[17]^ This could be attributed to a smaller sample size compared to ours, allowing for greater discrimination accuracy in the diagnostic values of the evaluated CDR. To explain the significant difference in the performance of the Geneva score in our population, it was noted that the Le Gal study had a lower proportion of patients with recent surgery or fracture (6.9% in the Le Gal group vs 23% in our population). This difference is attributed to our center receiving a high proportion of patients with these characteristics. A study by Miniati et al in a population with a PE prevalence of 43% reported an AUC of 0.54 for the Geneva score, with over 30% having recent surgery or fracture, supporting our hypothesis.^[18]^ This presents a potential cause for the disparities in our study and suggests an opportunity for a prospective study to assess the application and diagnostic performance of the evaluated CDR in this population.

In this study, low S was documented for each independently evaluated variable (all below 50% except age over 65 years), deviating from expectations for a clinical prediction rule and differing from findings in other studies,^[8,9]^ where S ranged from 80% to 99%, respectively. Additionally, in this study, the AUC values for the original and simplified Geneva scores were similar (0.58), with no statistically significant difference between the 2 (Table 5). This aligns with validations in 2 previous studies^[7,15]^ and suggests there is no significant difference in applying these CDRs in estimating the pretest probability of experiencing PE.

This test has historically diminished in importance compared to the Wells and PESI tests for assessing the likelihood of PE. With the documented findings in our studied population and the validation performed for a Colombian population,^[17]^ its application loses even more validity as a clinical decision rule due to its low ability to evaluate patients with the disease and discriminate between sick and non-sick individuals. However, it is essential to consider the possibility that a higher proportion of patients with a recent history of trauma or surgery may be associated with these poor results in our population. Therefore, a prospective study could be conducted to increase statistical power, decentralize the study, and evaluate the external validity of both scales in a sample of the Colombian population. This study could also include nonclinical variables that might enhance both S and E of the scale or, alternatively, associate it with other characteristics that also indicate pretest probabilities for pulmonary embolism. However, it is important to highlight that clinical judgment in the diagnosis of this condition is of great significance. The clinician’s experience and suspicion based on it can outperform clinical decision rules.^[19]^

Limitations of this study include its retrospective analysis, where only patients with CTPA or suspected PE were included in the statistical analysis, introducing a selection bias akin to spectrum purity. Although prospective studies offer clinical utility and outcomes aligned with generating new hypotheses, the single-center nature of our study may limit its external validity. On the positive side, this study has a sample that meets the requirements for scale validation corrected by Yates, enhancing the robustness and consistency of the study by finding convergent consistency between the 2 scales.

## 5. Conclusions

The application of standardized clinical decision-making tools, such as both the original and simplified Geneva scales, has been prevalent over time to predict the occurrence of PE. This study focused on the validation of both scales in this population, revealing results different from those documented in previous studies. There is no significant S or E that would make it a standalone management scale to determine whether a patient has PE. Despite having similar diagnostic performance, as evidenced in the ROCs, both scores exhibit nearly negligible discriminatory capacity. Therefore, it is imperative to explore other clinical prediction rules as part of the array of possibilities that can be employed to enhance the pretest probability for an accurate diagnosis. From a statistical standpoint, there are no differences between the original Geneva score and the simplified version, so the use of either does not affect their performance. However, the simplified scale holds an advantage due to its easier score calculation and subsequent patient classification.

## Acknowledgments

We would like to acknowledge the Universidad de La Sabana in this work.

## Author contributions

**Conceptualization:** Cristian López-Vega, Michel Pérez-Garzón, Leonora Ortiz-García-Herreros, Alirio Bastidas-Goyes.

**Data curation:** Michel Pérez-Garzón, Alirio Bastidas-Goyes.

**Formal analysis:** Cristian López-Vega, Michel Pérez-Garzón, Leonora Ortiz-García-Herreros, Alirio Bastidas-Goyes, Estefan Ramos-Isaza, Manuel Aramendiz-Narvaez, Henry Robayo-Amortegui.

**Funding acquisition:** Michel Pérez-Garzón, Alirio Bastidas-Goyes.

**Investigation:** Michel Pérez-Garzón, Leonora Ortiz-García-Herreros, Alirio Bastidas-Goyes.

**Methodology:** Michel Pérez-Garzón.

**Project administration:** Cristian López-Vega, Michel Pérez-Garzón, Leonora Ortiz-García-Herreros.

**Supervision:** Cristian López-Vega, Michel Pérez-Garzón, Alirio Bastidas-Goyes.

**Validation:** Cristian López-Vega, Michel Pérez-Garzón, Leonora Ortiz-García-Herreros, Alirio Bastidas-Goyes, Henry Robayo-Amortegui.

**Visualization:** Cristian López-Vega, Michel Pérez-Garzón, Leonora Ortiz-García-Herreros, Alirio Bastidas-Goyes, Estefan Ramos-Isaza, Manuel Aramendiz-Narvaez, Henry Robayo-Amortegui.

**Writing – original draft:** Cristian López-Vega, Michel Pérez-Garzón, Leonora Ortiz-García-Herreros, Alirio Bastidas-Goyes, Estefan Ramos-Isaza, Manuel Aramendiz-Narvaez, Henry Robayo-Amortegui.

**Writing – review & editing:** Cristian López-Vega, Michel Pérez-Garzón, Leonora Ortiz-García-Herreros, Alirio Bastidas-Goyes, Estefan Ramos-Isaza, Manuel Aramendiz-Narvaez, Henry Robayo-Amortegui.
